# Carbon Nanotube-Induced Pulmonary Granulomatous Disease: *Twist*1 and Alveolar Macrophage M1 Activation

**DOI:** 10.3390/ijms141223858

**Published:** 2013-12-06

**Authors:** Barbara P. Barna, Isham Huizar, Anagha Malur, Matthew McPeek, Irene Marshall, Mark Jacob, Larry Dobbs, Mani S. Kavuru, Mary Jane Thomassen

**Affiliations:** 1 Division or Pulmonary, Critical Care & Sleep Medicine, East Carolina University, Brody Medical Sciences Building, 600 Moye Blvd. Rm. 3E-149, Greenville, NC 27834, USA; E-Mails: barnab@ecu.edu (B.P.B.); isham.huizar@ttuhsc.edu (I.H.); malura@ecu.edu (A.M.); mcpeekm07@students.ecu.edu (M.M.); marshalli@ecu.edu (I.M.); jacobm@ecu.edu (M.J.); mani.kavuru@jefferson.edu (M.S.K.); 2 Department of Pathology, East Carolina University, Brody Medical Sciences Building, 600 Moye Blvd. Rm. 7S-10, Greenville, NC 27834, USA; E-Mail: dobbsl@ecu.edu

**Keywords:** *Twist*1, alveolar macrophages, carbon nanotubes, sarcoidosis

## Abstract

Sarcoidosis, a chronic granulomatous disease of unknown cause, has been linked to several environmental risk factors, among which are some that may favor carbon nanotube formation. Using gene array data, we initially observed that bronchoalveolar lavage (BAL) cells from sarcoidosis patients displayed elevated mRNA of the transcription factor, *Twist*1, among many M1-associated genes compared to healthy controls. Based on this observation we hypothesized that *Twist*1 mRNA and protein expression might become elevated in alveolar macrophages from animals bearing granulomas induced by carbon nanotube instillation. To address this hypothesis, wild-type and macrophage-specific peroxisome proliferator-activated receptor gamma (PPARγ) knock out mice were given oropharyngeal instillation of multiwall carbon nanotubes (MWCNT). BAL cells obtained 60 days later exhibited significantly elevated *Twist*1 mRNA expression in granuloma-bearing wild-type or PPARγ knock out alveolar macrophages compared to sham controls. Overall, *Twist*1 expression levels in PPARγ knock out mice were higher than those of wild-type. Concurrently, BAL cells obtained from sarcoidosis patients and healthy controls validated gene array data: qPCR and protein analysis showed significantly elevated *Twist*1 in sarcoidosis compared to healthy controls. *In vitro* studies of alveolar macrophages from healthy controls indicated that *Twist*1 was inducible by classical (M1) macrophage activation stimuli (LPS, *TNFα*) but not by IL-4, an inducer of alternative (M2) macrophage activation. Findings suggest that *Twist*1 represents a PPARγ-sensitive alveolar macrophage M1 biomarker which is induced by inflammatory granulomatous disease in the MWCNT model and in human sarcoidosis.

## 1. Introduction

Pulmonary granulomas may appear in infectious or inflammatory disorders but may also be associated with environmental agents such as carbon nanotubes. Production of carbon nanomaterials for consumer products is expanding in worldwide commerce [1] and is an area of environmental concern. Combustion-generated multiwall carbon nanotubes (MWCNT) or nanoparticles may also be detectable in non-manufacturing environments, for example in vapors from diesel fuel, methane, propane and natural gas [2]. Data from experimental animal models illustrate the potential of carbon nanotubes to induce inflammatory changes, fibrosis, or granulomas [3–6].

In order to explore pathophysiologic mechanisms of granuloma formation and persistence, we developed a carbon nanotubes model of chronic granulomatous disease [7]. This novel murine model of MWCNT-elicited chronic granulomatous disease exhibits many similarities to the pathology of sarcoidosis, a prototypical human granulomatous disease of unknown etiology [8]. Sarcoidosis has been linked to some environmental risk factors that favor carbon nanotube formation in ambient air. Examples include exposure to wood-burning stoves, fireplaces, or firefighting [9–12]. Chronic granulomatous inflammation is prominent in the MWCNT model together with classically activated (M1) alveolar macrophages that over-express a number of proinflammatory genes [8]. In this model, granulomas persist out to 90 days, in contrast to previous sepharose bead models in which granulomas resolve within three weeks [13].

Sarcoidosis is characterized by marked elevation of *T Helper 1* (*Th1*) genes such as *interferon gamma* (*IFNγ*) and *IL-12* [14,15]. In such a milieu, alveolar macrophages appear classically activated (M1) and are major producers of the M1-associated gene, *TNFα* [16]. The transcription factor, peroxisome proliferator-activated receptor gamma (PPARγ) is deficient in sarcoidosis alveolar macrophages compared to healthy controls, while the pro-inflammatory regulator, nuclear factor kappa B (NF-κB), is activated [17]. Healthy alveolar macrophages, unlike macrophages residing in other organs, express constitutively high levels of PPARγ, suggesting a unique role for PPARγ in maintaining lung homeostasis [18]. PPARγ, a well-studied regulator of glucose and lipid metabolism, is also a potent down-regulator of many pro-inflammatory pathways [19]. Recently, we found that macrophage-specific PPARγ deficiency exacerbated MWCNT-induced inflammation and granuloma formation, suggesting that PPARγ may also function as a negative regulator of chronic granulomatous disorders [20].

*Twist* proteins (*Twist*1 and *Twist*2) are basic helix-loop-helix (bHLH) transcription factors present in many cell types and first recognized as important regulators of embryonic mesenchymal development [21,22]. Interestingly, *Twist*1 expression is upregulated by NF-κB activation [23]. We initially noted elevated *Twist*1 expression in sarcoidosis after analyzing gene array data from sarcoidosis and healthy control bronchoalveolar lavage (BAL) cells. We hypothesized that granulomatous disease might be a causative factor in *Twist*1 expression and utilized our MWCNT model to explore this issue. Further, we hypothesized that *Twist*1 would be elevated by M1 but not an inducer of alternative (M2) stimuli. Findings confirmed our hypothesis by indicating that induction of *inflammatory granulomatous disease elevates Twist1* gene and protein expression in alveolar macrophages and that elevated *Twist*1 expression is associated with M1 activation.

## 2. Results and Discussion

### 2.1. Sarcoidosis Patients Display an M1 Profile in Bronchoalveolar Lavage

Microarray results indicated a prevalence of significantly upregulated M1 genes in sarcoidosis BAL as anticipated, based upon previous reports citing elevated *IFNγ* pathways in sarcoidosis lung [24] (Table 1). A sample of M2 associated genes showed no significant elevation from healthy control levels with the exception of CCL2 which has been noted previously in sarcoidosis [25,26]. Data also confirmed our previous report showing elevated *IFNγ* in sarcoidosis BAL cells [27], as well as other reports of elevated *STAT*1 [24,28]. *IL-12*, which is also elevated in sarcoidosis lung [29,30], promotes *STAT*4 activation and *Th1* development [31,32]. M1 associated *IFNγ* inducible chemokines (*CXCL* 9, 10, 11 and *CCL*5) were also elevated as previously reported [26,33,34].

### 2.2. *Twist*1 is Elevated in Sarcoidosis Alveolar Macrophages

**Figure 1.**
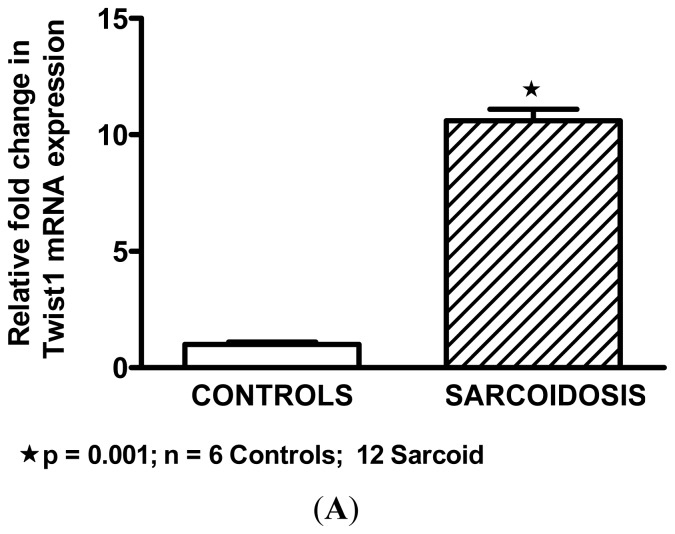

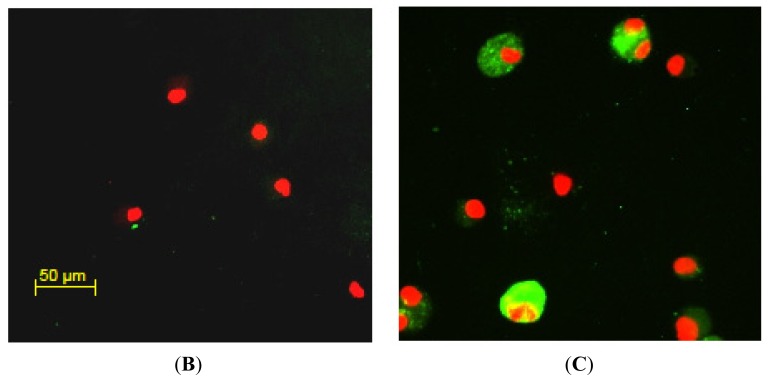
(**A**–**C**). *Twist*1 in human alveolar macrophages. *Twist*1 mRNA expression is intrinsically elevated in alveolar macrophages of sarcoidosis patients compared to healthy controls (**A**); Immunostaining did not detect *Twist*1 protein expression in healthy control alveolar macrophages (**B**) but *Twist*1 protein appears in alveolar macrophages of sarcoidosis patients; scale bar = 50 μm (**C**). Cells were counterstained with propidium iodide to highlight nuclei (same magnification as Figure 1B). Images are representative of findings from three patients and three controls.

Microarray studies also indicated a 5.1-fold increase in *Twist*1 (Probe set ID# 213943_at) expression in BAL cells from sarcoidosis patients compared to healthy controls. The false discovery rate was significantly low (0.04%). QPCR validated array data and demonstrated significant elevation of *Twist*1 expression in sarcoidosis patient BAL cells compared to healthy controls (Figure 1A). This observation was confirmed by immunostaining. No *Twist*1 staining was detectable in alveolar macrophages of healthy controls (Figure 1B). Evaluation of *Twist*1 protein in sarcoidosis BAL cells (Figure 1C) indicated elevated *Twist*1 in alveolar macrophages.

### 2.3. Macrophage M1 Inducers Upregulate *Twist*1

**Figure 2.**
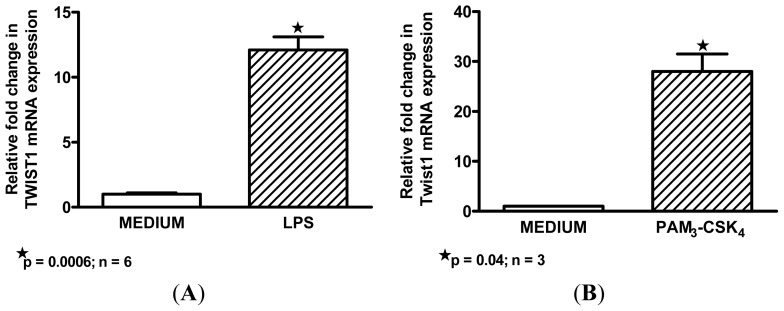

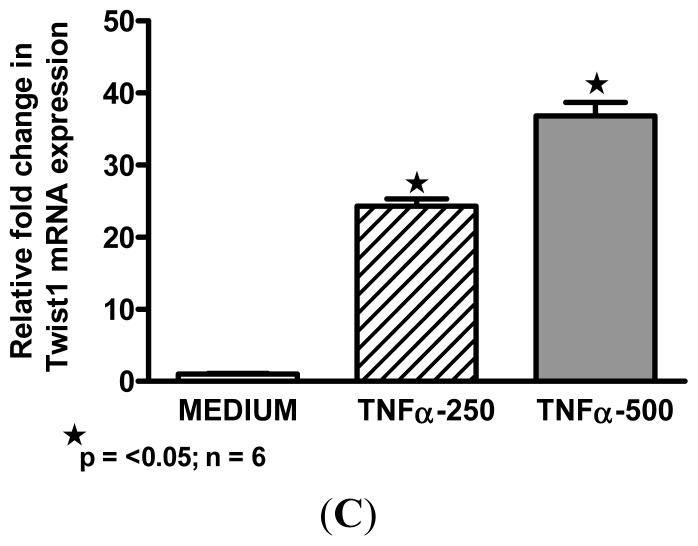
(**A**–**C**). Upregulation of *Twist*1 mRNA is found in healthy control alveolar macrophages exposed *in vitro* to M1 activators, LPS (**A**); PAM3-CSK4 (**B**); and *TNFα* (**C**).

Because of the striking M1 profile of sarcoidosis BAL cells, we hypothesized that *Twist*1 might also be an M1 biomarker. To address this issue, we obtained BAL cells from healthy individuals and cultured alveolar macrophages *in vitro* with each of three classic M1 inducers: the TLR4 ligand, LPS; TLR1-2 ligand, PAM3-CSK4; and the pro-inflammatory cytokine, *TNFα* [35,36]. Results indicated that all three reagents significantly upregulated alveolar macrophage *Twist*1 expression compared to control medium alone (Figure 2A–C). Cultures (*n* = 4) with the M2-associated inducer, *IL-4*, did not result in elevated *Twist*1 (data not shown).

### 2.4. *Twist*1 Is Elevated in Alveolar Macrophages from MWCNT-Granuloma Bearing Mice

**Figure 3.**
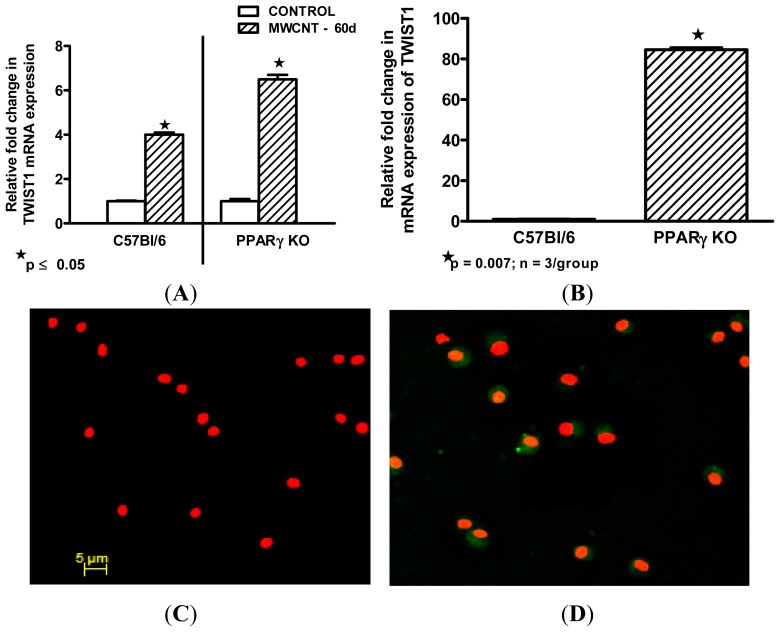
*Twist*1 in murine alveolar macrophages. Bronchoalveolar lavage (BAL) cells were obtained from C57Bl/6 wild-type and macrophage specific PPARγ KO mice at 60 days after oropharyngeal instillation of MWCNT. At this time point, all animals have extensive granulomatous disease in the lung [7,20]. *Twist*1 mRNA is elevated in BAL cells from MWCNT-treated wild-type (*n* = 7) mice *versus* sham controls (*n* = 6) as well as in MWCNT-treated macrophage specific PPARγ KO mice (*n* = 11) compared to sham controls (*n* = 7) (**A**); *Twist*1 mRNA is intrinsically elevated in BAL from untreated PPARγ KO mice compared to untreated wild-type controls (**B**); Immunostaining of alveolar macrophages from wild-type C57Bl/6 mice does not detect *Twist*1 protein after sham-treatment. Scale bar = 5 μm. (**C**); but shows elevated *Twist*1 protein after MWCNT instillation (same magnification as Figure 3C) (**D**).

To determine if granulomatous disease was a factor in *Twist*1 upregulation, wild-type C57Bl/6 mice and macrophage specific PPARγ knock out mice were instilled with MWCNT and euthanized 60 days later. By 60 days, pulmonary granulomas containing MWCNT are prominent in this model [20]. Macrophage specific PPARγ knock out mice were included because our previous report had observed increased granulomas in this group [20]. In addition our sarcoidosis studies had indicated PPARγ deficiency in sarcoidosis alveolar macrophages [17,27]. Compared to sham controls, significant elevation of BAL cell *Twist*1 mRNA expression was observed after MWCNT-treatment in both C57Bl/6 wild-type (4.0 ± 0.1 fold change) and macrophage specific PPARγ knock out (6.5 ± 0.2 fold change) mice (Figure 3A). In untreated mice, *Twist*1 mRNA expression was intrinsically elevated in PPARγ knock out mice compared to wild-type (Figure 3B). Examination of immunostained BAL cytospins from wild-type mice also indicated elevation of *Twist*1 protein after MWCNT treatment (Figure 3D) compared to sham-treated animals (Figure 3C).

### 2.5. Discussion

Findings suggest that alveolar macrophage *Twist*1 expression is upregulated by inflammatory granulomatous disease and by PPARγ deficiency. Our previous studies have shown that PPARγ becomes deficient in alveolar macrophages from MWCNT-treated wild-type mice [20]. The coincidence of elevated *Twist*1 and PPARγ deficiency in alveolar macrophages from macrophage-specific PPARγ null mice, MWCNT-instilled wild-type mice, and PPARγ-deficient sarcoidosis patients is an intriguing observation not previously reported. In fibroblasts and macrophages, *Twist* expression has been reported to be promoted by *TNFα* [23,37], and our results support those findings. Many studies have cited evidence for elevated *TNFα* in sarcoidosis patients [38–40]. *TNFα* is also produced in the MWCNT granuloma model [7]. Thus the inflammatory milieu of both sarcoidosis and MWCNT granulomatous disease contains inducers of *Twist*1 expression. *Twist*1 has also been observed in fibroblasts and epithelial cells within fibrotic foci from lung tissues of patients with idiopathic pulmonary fibrosis [41,42]. Further, *Twist*1 expression in these cells could be induced *in vitro* by pro-fibrotic growth factors or viral infection, respectively [41,42]. Collectively, such results suggest that cellular patterns of *Twist*1 expression may vary with disease pathology.

The current results also suggest that *Twist*1 may represent an M1 marker of alveolar macrophage activation. Classical (M1) activation can be driven by TLR ligands (reviewed in [36]). Both TLR ligands LPS and PAM3-CSK4 significantly increased *Twist*1 in alveolar macrophages from healthy donors. LPS appears to induce healthy alveolar macrophages to express an M1 phenotype with a transcription factor scenario (reduced PPARγ together with elevated NF-κB and *Twist*1) that resembles the phenotype of sarcoidosis alveolar macrophages [17,27] as well as that of MWCNT-instilled mice [20]. It should be noted that PPARγ is also an activator of alternative (M2) macrophage activation [43]. Thus the reduction of alveolar macrophage PPARγ noted in both sarcoidosis and MWCNT-instilled mice would most likely favor a M1 phenotype.

The functional consequences of *Twist*1 expression in alveolar macrophages remain to be explored. *Twist*1, a transcription factor that plays a vital role in formation of embryonic mesenchymal tissue, also appears in many types of mature cells [21,22]. Studies of *Twist*1 in mature cells are few and none have focused on alveolar macrophages. Investigations of rat lung fibroblasts indicate that *Twist*1 overexpression protects cells against apoptosis [41,42]. Other evidence suggests that *Twist* proteins may be NF-κB regulators. Deficiency or haplo-insufficiency of *Twist* proteins produces a lethal systemic inflammatory syndrome [23]. *Twist* expression is upregulated by NF-κB activation and *Twist* proteins may provide negative feedback by blocking p65/RelA-mediated transactivation, thus repressing NF-κB-dependent cytokine production [23]. Our previous findings indicate that NF-κB-related pro-inflammatory cytokines are elevated in MWCNT-instilled mice, thus suggesting a setting with both NF-κB and *Twist*1 upregulation [7,20]. Our present data support a link between *Twist*1 and the M1 macrophage activation phenotype in alveolar macrophages. The functional significance of alveolar macrophage *Twist*1 expression in MWCNT-induced granulomatous disease, however, must be determined in future studies.

## 3. Experimental Section

### 3.1. Human Study Population

Sarcoidosis subjects (*n* = 23) were recruited from patients undergoing routine clinical evaluation for initial diagnosis (12) or confirmation (11) of sarcoidosis (Table 2). None had Löfgren’s syndrome [44]. All diagnoses were confirmed by pulmonary histology demonstrating non-necrotizing granulomas in the absence of infection or other etiologies.

The healthy control group (*n* = 27) was composed of individuals with no history of lung disease and no medication usage at time of bronchoscopy (Table 2). These individuals volunteered to undergo bronchoscopy as part of an Institutional Review Board-approved research program (Greenville, NC, USA). The protocol was approved by the East Carolina University Institutional Review Board (Greenville, NC, USA) and written informed consent was obtained from all patient and control subjects.

### 3.2. Human Cell Collection and Culture

Bronchoalveolar lavage (BAL) cells were collected by fiberoptic bronchoscopy as previously described [17]. Differential cell counts were obtained from cytospins stained with modified Wright’s stain (Fisher Scientific, Kalamazoo, MI, USA). Mean viability of lavage cells was greater than 95% as determined by trypan blue dye exclusion. For culture, BAL cells were plated into 24-well plates (300,000 alveolar macrophages/well) or chamber slides (60,000 cells/well) in RPMI 1640 medium supplemented with 5% human AB serum (Gemini, Calabasas, CA, USA), l-glutamine, and antibiotics as described previously [45]. Adherence purified alveolar macrophages (60 min) were cultured for 24 h in medium alone, or medium supplemented with LPS (*Salmonella typhimurium*, Sigma, St. Louis, MO, USA) (500 ng/mL), PAM3-CSK4 (50 ng/mL) (Fisher Scientific, Waltham, MA, USA), *TNFα* (250–500 units/mL), or IL-4 (10 ng/mL) (R & D Systems, Minneapolis, MN, USA). Yields of patient or control BAL cells were not sufficient to allow performance of all experiments on each individual specimen.

### 3.3. Immunocytochemistry

Immunocytochemistry was performed on cytospin preparations from freshly isolated human or murine alveolar macrophages for *Twist*1 basal expression levels (Santa Cruz Biotech, Santa Cruz, CA, USA). Slides were fixed with 4% paraformaldehyde–PBS, then permeabilized with Triton X-100 and stained with anti-TWIST1 antibody (C-17, Santa Cruz, CA, USA) at 1:500 dilution, followed by Alexa conjugated goat anti rabbit IgG (Invitrogen, Carlsbad, CA, USA). Murine cells were stained similarly using anti-TWIST1 (H-81, Santa Cruz, CA, USA). Slides were counter stained with Propidium Iodide (PI) (Vector Laboratories, Burlingame, CA, USA) to facilitate nuclear localization.

### 3.4. Microarray Analysis

Microarray analyses of BAL cells from 12 sarcoidosis patients not on steroids and 10 healthy control individuals were carried out by Expression Analysis, Inc. (Durham, NC, USA) using the Affymetrix Human Genome GeneChip U133A 2.0 Array Plate (Affymetrix, Santa Clara, CA, USA). This array plate measures 14,500 well-characterized genes per sample. Total RNA was extracted from BAL samples and RNA quality was verified by the Agilent 2100 bioanalyzer (Agilent Technologies, Santa Clara, CA, USA). Amplified biotin-labeled cRNA was generated from 2 μg of total RNA. Fragmented cRNA was hybridized to gene chip arrays (Affymetrix, Santa Clara, CA, USA) according to the manufacturer’s instructions, then stained, washed and scanned. Principal component analysis (PCA) was conducted on all genes. Gene expression differences of patients *versus* controls were assessed by means of Student’s *t*-test, with a false discovery rate (FDR) correction for multiple testing (R Bioconductor, R Series, Seattle, WA, USA). Genes with a false discovery rate ≤0.05 and a fold change ≥2 were considered differentially expressed.

### 3.5. Quantitative mRNA Expression

Total RNA was extracted from human or murine cells by RNeasy protocol (Qiagen, Valencia, CA, USA). Expression of mRNA was determined by real time qPCR using the ABI Prism 7300 Detection System (TaqMan; Applied Biosystems, Foster City, CA, USA). Primer-probe sets for *TWIST1* and a housekeeping gene (*GAPDH*) were obtained from Qiagen, Germantown, MD, USA. Data were expressed as a fold change in mRNA expression compared to control values [46].

### 3.6. Murine MWCNT Model

All studies were conducted in conformity with Public Health Service (PHS) Policy on humane care and use of laboratory animals and were approved by the institutional animal care committee. C57BL/6J wild-type mice and macrophage specific PPARγ KO conditional mice received an oropharyngeal instillation of MWCNT (100 μg in surfactant/PBS) after sedation with isofluorane. Sixty days after sham (surfactant in PBS) or MWCNT treatment, mice were sacrificed, and bronchoalveolar lavage (BAL) or lungs were harvested for further analysis as previously described [7].

### 3.7. Characterization of Carbon Nanotubes

MWCNTs (catalogue number 900–1501, lot GS1801) were obtained from SES Research (Houston, TX, USA). Scanning electron microscopy (Hitachi, Tokyo, Japan) was used to determine the structural characteristics of MWCNTs. Nitrogen adsorption studies were carried out using a physisorption analyzer (ASAP 2010; Micromeritics, Norcross, GA, USA). Full details of MWCNT characterization have been described previously [7].

### 3.8. Statistical Analyses

QPCR data were analyzed by one-way analysis of variance (ANOVA) and Tukey’s test using Prism software (GraphPad, Inc., San Diego, CA, USA). Data from *in vitro* studies were evaluated by Students *t*-test.

## 4. Conclusions

Current findings suggest that alveolar macrophages exhibit *Twist*1 expression when an M1 stimulatory milieu, as found in chronic granulomatous disease, dysregulates pulmonary homeostasis. Application of the MWCNT granuloma model will be a useful tool to explore the potential interactions of *Twist*1 with the transcription factors, NF-κB and PPARγ in subsequent studies of pulmonary granulomatous disease.

## Figures and Tables

**Table 1.** M1 and M2-associated genes in Sarcoidosis gene array samples.

Gene Symbol	Gene Name	Fold Change
**M1-associated Genes**		

*IFNγ*	*Interferon-gamma*	7.13
*IL6*	*Interleukin 6*	2.83
*IL18R1*	*Interleukin 18 receptor 1*	4.59
*IL12Rb2*	*Interleukin 12 receptor, beta 2*	6.32
*STAT1*	*Signal transducers and activators of transcription 1*	2.05
*STAT4*	*Signal transducers and activators of transcription 4*	3.82
*CXCL11*	*Interferon inducible T cell α chemoattractant (I-TAC)*	3.93
*CXCL10*	*Interferon inducible protein 10, (IP-10)*	3.48
*CXCL9*	*Monokine induced by interferon*γ*, (MIG)*	4.64
*CCL5*	*Chemokine (C-C motif) (RANTES)*	5.77

**M2-associated Genes**		

*IL10*	*Interleukin 10*	NS *
*IL1RA*	*Interleukin-1 receptor antagonist*	NS *
*CD36*	*Member of the class B scavenger receptor*	NS *
*MMP2*	*Matrix metalloproteinase 2*	NS *
*MMP7*	*Matrix metalloproteinase 7*	NS *
*CCL24*	*Chemokine (C-C motif) ligand 24*	−5.35
*CCL2*	*Chemokine (C-C motif) ligand 2*	2.71

* NS = not significantly different from healthy control.

**Table 2.** Demographics of Patients and Control Subjects.

Characteristics	Sarcoidosis	Healthy Controls
	
	(*n* = 23)	(*n* = 27)
**Age (year)**	44.8 ± 2.6	32.4 ± 1.4
**Gender**	15F/8M	18F/9M
**Self-reported race**	21AA/2C	14AA/12C/1AI
**Smokers**	0 (10 exsmokers)	0 (1 exsmokers)
**FVC % predicted**	80.6 ± 3.8	–
**CXR stage: 0–1**	6*	–
**CXR stage: 2–4**	16	–
**BAL Macrophages (%)**	83.3 ± 2.8	95.5 ± 0.6
**BAL Lymphocytes (%)**	15.2 ± 2.8	4.0 ± 0.6
**BAL PMNs (%)**	1.6 ± 1.0	0.4 ± 0.2
**Main treatment indication(s) at time of bronchoscopy**	Not treated (11)	–
	pulmonary (9)	
	multiorgan-(dermal, ocular) (3)	
**Organ involvement**	Lung (23)	–
	Multiorgan (12) **	

* no radiographs (1);

** ocular, cardiac, dermal, neuro, hepatic, splenic.
